# Bisbenzylisoquinoline Alkaloids of *Cissampelos Sympodialis* With in Vitro Antiviral Activity Against Zika Virus

**DOI:** 10.3389/fphar.2021.743541

**Published:** 2021-09-15

**Authors:** Poliana Gomes da Silva, Aventino H. Fonseca, Malu P. Ribeiro, Taizia D. Silva, Cristiane F. F. Grael, Lindomar J. Pena, Tania M. S. Silva, Eduardo de Jesus Oliveira

**Affiliations:** ^1^ Department of Virology, Aggeu Magalhães Institute (IAM), Fiocruz, Recife, Brazil; ^2^ Department of Pharmacy, Federal University of Jequitinhonha and Mucuri Valleys, Diamantina, Brazil; ^3^ Laboratório de Bioprospecção Fitoquímica, Department of Chemistry, Federal Rural University of Pernambuco, Recife, Brazil

**Keywords:** *Cissampelos sympodialis*, Zika virus, antiviral activity, warifteine, methylwarifteine, bisbenzylisoquinoline alkaloids

## Abstract

In search of new antiviral compounds against Zika virus we conducted a bioassay-guided fractionation of bisbenzyilisoquinoline alkaloids isolated from *Cissampelos sympodialis* (Menispermaceae), a medicinal plant species endemic to Brazil. Six subfractions were obtained from a tertiary alkaloidal fraction of the rhizomes (TAFrz) using preparative high-performance liquid chromatography. All the subfractions were tested against Zika virus-infected Vero cells as the cellular model to evaluate cytotoxicity and antiviral effective concentrations. The results showed that three of the six TAFrz subfractions tested were active. The most active ones were the subfraction 6 (that consisted of the alkaloids methylwarifteine and warifteine present as a mixture at a ratio of 8.8:1.2 respectively) and the subfraction 5, that was later identified as warifteine, the major tertiary alkaloid of this species. Warifteine was able to significantly reduce virus titer in Zika virus-infected Vero cells with an IC_50_ of 2.2 μg/ml and this effect was selective (selectivity index, SI = 68.3). Subfraction 6 had an IC_50_ = 3.5 μg/ml and was more cytotoxic than pure warifteine, with SI = 6.14. Fraction 5 and fraction 6 were more potent in decreasing the viral titer of Zika virus-infected Vero cells than 6-methylmercaptopurine riboside (IC_50_ = 24.5 μg/ml and SI = 11.9), a mercaptopurine riboside with ZIKV antiviral activity used as a positive control. Our data demonstrate that alkaloids of the bisbenzylisoquinoline type may be explored as new antiviral agents or as an useful pharmacophore for investigating ZIKV antiviral activity.

## Introduction

*Cissampelos sympodialys* Eichl. (Menispermaceae) is a plant species endemic to Brazil. The infusion of hot water from the leaves and bark of the root of *C. sympodialis* is widely used in indigenous and popular medicine to treat a number of conditions such as bronchitis and asthma (Bezerra-Santos et al., 2006; Agra et al., 2007). Based on this empirical knowledge, it became important to carry out non-clinical studies with the objective of investigating the possible pharmacological activities of this plant (Melo et al., 2003). *Cissampelos sympodialis* have yielded a wealth of biological activities due to the immunomodulatory (Bezerra-Santos et al., 2006), spasmolytic (de Freitas et al., 1996) and anti-asthma properties (Bezerra-Santos et al., 2012) of its metabolites, attributed mainly to the bisbenzylisoquinoline alkaloids wariftein and methylwariftein (Mukherjee and Keifer 2003) and to the morphinandienone alkaloid milonine (de Freitas et al., 1995). Others alkaloids that were also isolated from this species include the tertiary bisbenzylisoquinoline base roraimine (de Lira et al., 2002), and the quaternary aporphinic alkaloid laurifoline (Dominguez et al., 1974; Marsaioli et al., 1979).

Despite the many biological activities that have been characterized for the bisbenzylisoquinoline alkaloids from *C. sympodialis*, the antimicrobial activity of these alkaloids have been less investigated. However, the antiviral activity of several other alkaloids of the bisbenzylisoquinoline type have been reported. These include the anti-HIV-1 effect of fangchinoline (Wan et al., 2012), the effect against Herpes Simplex Virus (HSV) of obamegine (Nawawi et al., 1999) and the anti-influenza effect of isotetrandrine (Zeng et al., 2006). The effects of *C. sympodialis* leaf extract and of isolated warifteine against Dengue virus was previously studied (Leite et al., 2016). More recently, our group reported the antiviral activity of alkaloids from *Cissampelos sympodialis* against serotype II Dengue virus. The Dengue virus is a well-known pathogen from the *flavivirus* family sharing many molecular and structural similarities with the Zika virus (Sukhralia et al., 2019), a virus that became a pathogen of public concern and an important public health issue in the last years.

Zika virus (ZIKV) was first isolated in 1947 in the Zika Forest in Uganda (Dick et al., 1952; Barrows et al., 2018). Between March 2015 to the end of January 2016, more than 20 countries reported outbreaks of ZIKV (Agumadu and Ramphul 2018). In November 2015, an accumulation of microcephaly cases in Brazil was associated with the ZIKV epidemic, and then the virus gained prominence in the world public health scenario (Barrows et al., 2018). Despite the strong of Zika virus infection during pregnancy and the development of severe neurological disorders, there are challenges associated with a causal relationship and the exact pathophysiological mechanism responsible for these disorders remains unclear (Chang et al., 2016). However, the causal link between ZIKV infection during pregnancy and microcephaly is strengthening with recent case control studies (Rocha et al., 2019). In February 2016, WHO classified the disease as emerging and of great concern to global public health due to its rapid and uncontrolled spread. Currently, the virus is considered endemic in 79 countries. (Sukhralia et al., 2019). Also, there is no effective vaccine or drug treatment for Zika virus, increasing the relevance and importance of searching for new antiviral agents. Given the previous results reporting the activity of bisbenzylisoquinoline alkaloids against different viral agents, and our work with *C. sympodialis* alkaloids with Dengue antiviral activity, we decided to investigate the activity of these substances against ZIKV using a bioassay-guided fractionation strategy.

## Materials and Methods

### Materials

HPLC-grade methanol and acetonitrile were purchased from Tedia Brazil (Tedia Brazil, RJ). All other solvents were reagent grade. Water was purified using a Millipore Milliq Direct water purification system (Millipore, Billerica, MA, United States). Deuterated chloroform and methanol were obtained fom Tedia Brazil (Tedia Brazil, RJ). C-18 solid-phase extraction cartridges (Discovery DSC-18 SPE, 5g/20 ml) was purchased from Sigma-Aldrich, Brazil.

### Plant Material and TAFrz Preparation

Rhizomes of *C. sympodialis* Eichl were obtained from cultivated specimens from the Medicinal Herb Garden of the Biotechnology Center (CBiotec), Federal University of Paraiba (UFPB) at João Pessoa, Paraíba Brazil (7.141632S, 34.846290W). A voucher specimen (AGRA 1476) was deposited at the Lauro Pires Xavier Herbarium, UFPB). The crude ethanol extract of the rhizomes was prepared by exhaustive extraction of the plant material (1,6 Kg of dried powdered rhizomes) with 70% ethanol (basified to pH = 10 with ammonium hydroxide) until negative Dragendorff test. The extract was concentrated under reduced pressure to afford 111 g of crude ethanolic extract of the rhizomes (EtOHrz). The tertiary alkaloid fraction of the ethanolic crude extract of rhizomes (TAFrz, 15.8 g) was obtained by conventional acid/base extraction as previously described (Marinho et al., 2013). The use of the plant material in this study was registered in the Brazilian National System for the Management of Genetic Heritage and Associated Traditional Knowledge under the protocol number A3016A6.

### HPLC Fractionation of TAFrz and Isolation of Alkaloids

The preparation of TAFrz subfractions and isolation of warifteine and methylwarifteine was achieved using a preparative High Performance Liquid Chromatography method. A high-pressure gradient HPLC system consisting of 3 LC-6AD solvent delivery module, a DGU-20A5 degasser unit, a CBM-20A controller and a SPD-M20A photodiode array detector was used (all from Shimadzu, Shimadzu, Japan). Separation was achieved using a PREP-ODS C-18 reversed phase column (250 × 20 mm, Shim-pack, Shimadzu, Japan) with solvent delivered at a flow rate of 12 ml/min. A gradient of water (A) and methanol (B) both basified to pH 8.0 with ammonium hydroxide was used: 0–7 min (65%B), 8–15 min (70%B), 16–28 min (80%B). Detection was at 278 nm. Using the described method 6 peaks/subfractions were collected. The solvent from these subfractions was removed using a solid phase (SPE) extraction method with Discovery DSC-18 SPE cartridges. Previous to sample application the cartridges were conditioned with 20 ml of methanol (4x5ml) followed by 20 ml of water using a vacuum manifold. Each subfraction collected using HPLC was diluted with water until methanol concentration was 10% or lower (v/v) before application into the cartridges to prevent sample breakthrough. Cartridges were then purged and dried by vacuum application and finally eluted into glass tubes using 4 × 5 mL of acetonitrile (MeCN). Samples were transferred to round-bottom flasks and the solvent was evaporated to dryness using a rotary evaporator under reduced pressure. The purity of collected peaks was evaluated using an analytical HPLC method with a Luna C-18 column (250 × 4.6mm, 5 µm particle diameter) and an isocratic mobile phase consisting of 40% MeOH and 60% H_2_O (v/v) delivered at a flow rate of 0.8 ml/min with UV detection at 278 nm. NMR spectra were acquired on a Bruker Avance 400 MHz spectrometer. All NMR spectra were run at 300 K from CDCl_3_ solutions with 0.5% CD_3_OD (∼0.6 ml). The chemical shifts were expressed in the δ (ppm) scale and were internally referenced to residual CHCl_3_.

### LC/MS Analysis

The results were obtained using a XEVO-G2XSQTOF mass spectrometer (Waters, Manchester, United Kingdom) which was connected to an ACQUITY UPLC system (Waters, Milford, MA, United States). The conditions for obtaining the data by UPLC-ESI-qTOF-MS/MS were according to Silva *et al.* (Silva et al., 2020).

### Cells and Viruses

Vero cells were grown in Dulbecco’s modified Eagle’s medium (DMEM) (Gibco, Carlsbad, CA, United States) supplemented with 10% inactivated fetal bovine serum (FBS) (Gibco), 2 mM L-glutamine (Gibco) and 100 U/mL penicillin/streptomycin (Gibco, Carlsbad, CA) and 2.5 μg/ml of amphotericin (Gibco, Carlsbad, CA, United States). The strain of ZIKV used was isolated from the blood of a patient with rash disease in the State of Pernambuco and is called ZIKV/*H. sapiens*/Brazil/PE243/2015 (GenBank: KX197192.1). ZIKV PE243 strain was stored at −80°C degrees until the virus propagation and titration on Vero cells through TCID50 (50% Tissue Culture Infectious Dose) Method. All the experiments and virus manipulation were done in a Biosafety Level 2 (BSL-2) facility with approval by the Brazilian National Committee on Biosafety (certificate CQB: 98/99) following all the necessary Brazilian legislation and according to the recommended biosafety rules.

### Cell Viability Assay

The toxicity of fractions and isolated alkaloids was tested on growing Vero cells using *in situ* mitochondrial reduction of the tetrazolium dye 3-(4,5- dimethylthiazol-2-yl)-2,5-diphenyltetrazolium bromide (MTT) (MTT was purchased from Sigma Aldrich, Brazil). Briefly, 24-h-plated Vero cells (1 × 10^4^ cells/well) in 96-well microplates were treated with increasing concentrations (0; 0.75; 1.5; 3.0 and 6.0 μg/ml) of TAFrz subfractions, warifteine or methylwarifteine diluted in dimethyl sulfoxide (DMSO) (DMSO was obtained from Labsynth, Brazil). The final DMSO concentration was equal or lower than 0.06% v/v, as this concentration did not induce cytotoxic effect on Vero Cells. After 120 h of incubation at 37°C in a 5% CO_2_ atmosphere, culture medium was removed and 50 μL of freshly prepared MTT solution (1 mg/ml) was added to each well. The microplate was then incubated for 4 h at 37°C in a CO_2_ incubator, and MTT formazan crystals were solubilized by adding dimethyl sulfoxide (DMSO). The optical density at 540 nm (OD540) was determined spectrophotometrically using the BioTekTM ELx800TM 96- well plate reader (BioTek Instruments Inc., Winooski, VT). Cell viability was calculated by subtracting the OD540 of treated cells from untreated cells. The 50% cytotoxic concentration of the cell culture (CC_50_) was defined as the extract concentration that reduced the cell viability by 50% when compared to untreated controls.; CC_20_ (the concentration of compound that caused a 20% reduction in absorbance) was defined as the safe concentration for antiviral screening. Results were expressed as mean ± SD of three independent replicates.

### Evaluation of Cytopathic Effect on Vero Cells

Vero cells were seeded in 24-well plates 1 day prior to infection at a density of 5×10^4^ cells/well. Cells were infected with ZIKV PE243 strain at a multiplicity of infection (MOI) of 0.1 and were incubated for 2 h at 37°C in 5% CO_2_. Following virus internalization, the viral inoculum was removed, cells were washed twice with DMEM and the supernatant was replaced with fresh medium containing 6MMPr as positive control or TAFrz subfractions, warifteine or methylwarifteine at their CC_20_ concentrations as determined in the cell viability assay. Controls included mock (non-infected) and infected non-treated cells (described as “ZIKV” group in Figure 2C). The evaluation of cytopathic effects included cell modifications such as loss of attachment (detaching), shrinkage (cell hypertrophy, growth restriction) and syncytia formation (clumping of adherent cells) as described by Alpuche-lazcano (Alpuche-lazcano et al., 2018) The cytopathic effect was evaluated up to 120 h post infection (hpi) using an inverted microscope (AE2000 binocular microscope, Motic, Hong Kong) and pictures were taken using a smartphone. At 120 hpi, the cell supernatant was harvested and was stored at −80°C until analysis.

### Antiviral Activity Assay (Viral Titration)

Viral titration was performed by the standard TCID_50_ method and was expressed as log_10_ TCID_50_/mL. Vero cells were cultivated in 96 well plates at the density of 1.10^4^ cells/well at 37°C in a 5% CO_2_ incubator 1 day prior to titration. Cell supernatants stored from the cytopathic effect assay (see item 2.6) were 10-fold serially diluted in DMEM and added to the cells, which were further incubated for 5 days at 37°C and 5% CO_2_. After this time, the cytopathic effect was evaluated on an inverted optical microscope. The CPE is defined for each well and the TCID_50_ is calculated using the method of Reed and Muench, 1938. The reduction of viral titer was expressed as log_10_ TCID_50_/mL.

### Statistical Analysis

Data are expressed as mean ± SD of three independent experiments unless otherwise stated. Differences in viral titer between treated and untreated infected cells were evaluated by one-way analysis of variance (ANOVA) with Dunnett test, using the software GraphPad Prism v.5.01 for Windows (GraphPad Software, La Jolla, CA). The 50% inhibitory concentration (IC_50_) was defined as the compound concentration required to reduce the ZIKV titer by 50% compared with the virus control. Values of CC_50_ and IC_50_ were calculated by non-linear regression using GraphPad Prism software v.8.0 (GraphPad Software, San Diego, CA). The selectivity index (SI), which represents the difference between cytotoxicity and antiviral activity, was obtained by calculating the ratio of the CC_50_ and IC_50_ values. Significance was considered when *p* < 0.05.

## Results and Discussion

### Fractions Obtained and Identification of Alkaloids

**FIGURE 1:**
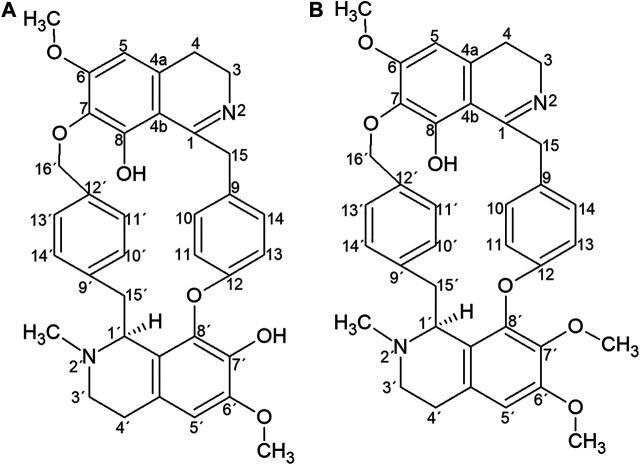
Chemical structure of warifteine **(A)** and methylwarifteine **(B)**.

Six fractions from preparative HPLC were collected from the total tertiary alkaloid fraction from the rhizomes (TAFrz). The yields of these fractions are shown in Table 1. We identified the alkaloids present in the most bioactive fractions as revealed by the antiviral assays. The identification of the alkaloids was based on a combination of ^1^H NMR spectroscopy and ultra-performance liquid chromatography coupled with high resolution time of flight electrospray mass spectroscopy (UPLC-ESI-qTOF-MS/MS). The full description of the identification of the alkaloids and associated spectroscopic data was previously published (da Silva et al., 2020). Briefly, chromatographic analysis of Fraction 5 resulted in a single peak in analytical HPLC. By comparison of the ^1^H NMR spectroscopy data and high resolution mass spectra of fraction 5 with published reference data (Snedden et al., 1970; Aguirre-Galvis, 1988; Mukherjee and Keifer 2003) the fraction was identified as warifteine (Figure 1), the main alkaloid reported to occur in the rhyzomes of *C. sympodialis*. Fraction 6 was identified as methylwarifteine by comparison of ^1^H NMR data with reference data (Mukherjee and Keifer 2003). However, when Fraction 6 was submitted to analysis by ultra performance liquid chromatography high-resolution time of flight tandem mass spectrometry analysis (UPLC-QTOF-MS^E^) it proved to consist of a mixture of warifteine and methylwarifteine (in the ratio of 87.5% methylwarifteine to 12.5% warifteine based on peak area by ultra-performance liquid chromatography with diode-array detector. Due to the low yield of sample 6 it was used in the antiviral experiments without additional purification.

**TABLE 1:** Yield of TAFrz and its subfractions.

Sample	Yield	Aspect
TAFrz	14.26%	Amorphous yellow powder
subfraction 01	0.010%	Amorphous brown powder
subfraction 02	0.033%	Amorphous brown powder
subfraction 03	0.044%	Amorphous dark yellow powder
subfraction 04	0.034%	Amorphous yellow powder
Subfraction 5 (Warifteine, WAR)	0.244%	Amorphous yellow powder
Subfraction 6	0.035%	Amorphous yellow powder

a Yield of TAFrz expressed in relation to the mass of crude EtOH extract.

b Yield of Fractions expressed in relation to the mass of TAFrz.

### Effect of Samples on Cell Viability

Table 2 shows the calculated CC_50_ and CC_20_ values for the samples tested. All values, including those of pure compounds, are expressed in the table as µg/mL to facilitate comparison between samples. Amongst the samples tested, the crude ethanolic extract of the rhizomes (EtOHrz) proved to be the most cytotoxic, with TAFrz as well as its subfractions exhibiting a lower toxicity to Vero cells. The fact that the ethanolic extract present a higher toxicity than fractions derived from it, may reflect the fact that the ethanol extract contains both polar and nonpolar substances, that could present a higher toxicity and are excluded when the acid-base liquid-liquid extraction is employed to prepare TAFrz. Warifteine, the major bisbenzylisoquinoline alkaloids from the rhizomes was also tested. The alkaloid showed a CC_50_ value similar to 6- methyl mercaptopurine riboside (6-MMPr), a thiopurine drug that was previously characterized as a West Nile virus and DENV-2 inhibitor (Lim et al., 2011), and that recently was shown by our group (de Carvalho et al., 2017) to have antiviral activity also against Zika virus (ZIKV). The calculated CC_20_ values were used for the antiviral assays as the maximal non-toxic concentration. Due to the observed toxicity of EtOHrz and the tendency of TAFrz subfractions to display lower toxicity to Vero Cells, we decided to test only TAFrz subfractions and warifteine for all subsequent experiments.

**TABLE 2:** Citotoxicity endpoints of the samples as determined by MTT assay on Vero cells.

Sample	Citotoxicity	
	CC_50_ (µg/ml)	CC_20_ (µg/ml)
EtOHrz	13.35	5.73
TAFrz	24.58	9.82
subfraction 01	19.72	7.12
subfraction 02	14.10	5.24
subfraction 03	23.24	9.58
subfraction 04	14.77	2.86
Subfraction 5 (Warifteine, WAR)	89.00	6.21
Subfraction 6	21.49	7.89
Positive Control (6MMPr)	86.00	17.88

### Antiviral Effect of Samples

**FIGURE 2:**
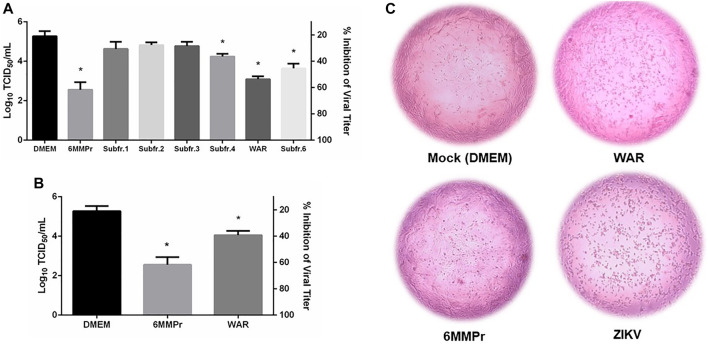
**(A)** and **(B)** Effect of warifteine (WAR) and TAFrz subfractions on virus titer. Vero cells were infected with a Multiplicity of Infection (MOI) of 0.1 of ZIKV for 2 h. The cell monolayer was then treated with the corresponding CC_20_ of each sample and incubated for 120h, and the supernatant collected and titrated using the TCID_50_ method. Values of concentration are given in µg/mL. Results are expressed as mean ± SD of 3 independent experiments. *p < 0.05 in relation to untreated Vero Cells (DMEM group). **(C)** Protection of Vero Cells from ZIKV-induced cytopathic effect by WAR and 5-MMPr. Mock (negative control) corresponds to non-infected cells treated only with DMEM. ZIKV (positive control) corresponds to cells infected with ZIKV and not treated.

Samples were tested for their capacity to decrease ZIKV viral titer in a culture of Vero cells. Figure 2A shows that three of TAFrz subfractions, were able to significantly (*p* < 0.05) decrease the viral titer when incubated with Vero cells for 120 h post-infection. The most potent sample was subfraction 5 (Warifteine) which at 10.49 μg/ml (corresponding to its CC_20_) was able to inhibit 80% of viral titer (Figure 2B), followed by subfraction 6 that was able to inhibit between 40–60% the viral titer and subfraction 4 that even though was less potent still demonstrated significant reduction in viral titer.

Figure 2C shows the effect of WAR in protecting Vero cells in culture against the cytopathic effect that follows ZIKV infection. Warifteine was able to prevent the morphological changes associated with progression of infection preserving the cell monolayer from shrinkage and clumping that characterizes the cytopathic effect, as can be seen in “ZIKV”, Figure 2C. The protective effect of WAR against ZIKV induced cytopathic effects reported here suggests that it can be mediated by anti-inflammatory or anti-oxidant properties. Lima et al., 2014 describes the anti-inflammatory effects of WAR through interference with neutrophil adhesion and migration, a common immune response in pathogenic infection. However, as reported by Leite et al., 2016, WAR and MeWAR were not able to decrease the production of TNF-α, IL-8 or macrophage migration inhibitory factor (MIF) in DENV-2 infected cells. This may demonstrate that besides the already known influence on cytokine secretion factors (Spelman et al., 2006) by herbal immunomodulators such as *Cissampelos sympodialis*, direct targets on the pathogen itself can also constitute a mechanism of action of these compounds. Recently our group reported the antiviral activity of bisbenzylisoquinoline alkaloids from *Cissampelos sympodialis* such as warifteine and methylwarifteine against dengue virus (da Silva et al., 2020). Indeed, it has been demonstrated that the use of other natural products, including alkaloids, were able to inhibit ZIKV and DENV infections by targeting the host cell at the virus-cell entry process or later at the viral replication complex (Gao et al., 2019; Fikatas et al., 2021).

It cannot be ruled out that the negative antiviral results of WAR against DENV reported in the work of (Leite et al., 2016) may reflect the fact that sample preparation involved the dissolution of the sample in hydrochloric acid, thus forming the alkaloid hydrochloride salt that is less permeable through cell membranesOur decision to use dimethyl sulfoxide as the solvent to dissolve our samples may explain these seemingly contradictory results, such as pointed out in a previously published work by our group (da Silva et al., 2020).

Table 3 summarizes the citotoxity and antiviral endpoints calculated for warifteine, and 6-MMPr. Warifteine was the most potent substance tested (with an IC_50_ of 2.00 μg/ml). Compared on a molar basis, warifteine is approximately 22 times more potent than 6-MMPr, used here as a positive control and has also a greater selectivity index. Fraction 6 (a mixture of methylwarifteine and warifteine in a ratio of 8.8:1.2) was also active (IC_50_ = 3.5 μg/ml) although less selective in its action than warifteine. It will be interesting to investigate in the future the antiviral activity of pure methylwarifteine which may be more potent than warifteine on a molar basis.

**TABLE 3:** Citotoxicity (CC_20_ and CC_50_), antiviral potency (IC_50_) and antiviral Selectivity Index (SI) for warifteine, methylwarifteine and 6-MMPr for infection of Vero cells with ZIKV.

	^d^CC_20_ (μg/ml)	^a^CC_50_ (μg/ml)	^b^IC_50_ (μg/ml)	^c^SI
Warifteine	10.49	150.3	2.2	68.3
Subfraction 6	7.89	21.49	3.5	6.14
6-MMPr	60.5	291	24.5	11.9

a CC_50_ (50% cytotoxic concentration) refers to compound concentration that caused a 50% reduction in viability.

b IC_50_ (50% inhibitory concentration) refers to compound concentration required to reduce viral titres by 50% compared with untreated controls.

c The Selectivity Index (SI) was calculated as the ratio of the CC_50_ to the IC_50_.

d CC_20_ (20% cytotoxic concentration) was taken as the non-toxic concentration to be employed in the antiviral assays.

Results are mean values of 3 independent experiments.

Taken together our data demonstrate that warifteine, the major alkaloid present in the tertiary alkaloidal fraction of *Cissampelos sympodialis* as well as methylwarifteine exhibit potent ZIKV *in vitro* antiviral effects. These results need to be interpreted as preliminary since we did not conduct a molecular characterization of these effects. Thus, further confirmation and characterization of the antiviral acitivity of warifteine and methylwarifteine needs to be done. However, these alkaloids were more potent than a riboside analog previously characterized as possessing antiviral activity against ZIKV. Despite the selectivity index of the alkaloids investigated in this study being lower than antiviral drugs that are currently used in therapy, our results indicate that these molecules can become important lead compounds for the development of potent and selective antiviral drugs against ZIKV infection or as pharmacological tools to explore new therapeutically useful targets in the pathogenesis of ZIKV infection.

## Data Availability

The original contributions presented in the study are included in the article/supplementary material, further inquiries can be directed to the corresponding author/s.
